# Identification of a Proteasome-Targeting Arylsulfonamide with Potential for the Treatment of Chagas’ Disease

**DOI:** 10.1128/AAC.01535-21

**Published:** 2022-01-18

**Authors:** Marta L. Lima, Lindsay B. Tulloch, Victoriano Corpas-Lopez, Sandra Carvalho, Richard J. Wall, Rachel Milne, Eva Rico, Stephen Patterson, Ian H. Gilbert, Sonia Moniz, Lorna MacLean, Leah S. Torrie, Carmine Morgillo, David Horn, Fabio Zuccotto, Susan Wyllie

**Affiliations:** a Division of Biological Chemistry and Drug Discovery, Wellcome Centre for Anti-Infectives Research, School of Life Sciences, University of Dundee, Dundee, United Kingdom; b Drug Discovery Unit, Wellcome Centre for Anti-Infectives Research, School of Life Sciences, University of Dundee, Dundee, United Kingdom

**Keywords:** drug discovery, proteasome, malic enzyme, drug target, mechanism of action, Chagas’ disease

## Abstract

Phenotypic screening identified an arylsulfonamide compound with activity against Trypanosoma cruzi, the causative agent of Chagas’ disease. Comprehensive mode of action studies revealed that this compound primarily targets the T. cruzi proteasome, binding at the interface between β4 and β5 subunits that catalyze chymotrypsin-like activity. A mutation in the β5 subunit of the proteasome was associated with resistance to compound 1, while overexpression of this mutated subunit also reduced susceptibility to compound 1. Further genetically engineered and *in vitro*-selected clones resistant to proteasome inhibitors known to bind at the β4/β5 interface were cross-resistant to compound 1. Ubiquitinated proteins were additionally found to accumulate in compound 1-treated epimastigotes. Finally, thermal proteome profiling identified malic enzyme as a secondary target of compound 1, although malic enzyme inhibition was not found to drive potency. These studies identify a novel pharmacophore capable of inhibiting the T. cruzi proteasome that may be exploitable for anti-chagasic drug discovery.

## INTRODUCTION

The protozoan parasite Trypanosoma cruzi is the etiological agent of Chagas’ disease, also known as American trypanosomiasis. This zoonotic disease is endemic in Latin American countries, where an estimated 6 to 7 million individuals across 21 countries are infected. Due to migration from countries of endemicity, Chagas’ disease is now a worldwide problem, with hundreds of thousands of infected individuals now residing in the United States and Europe. The acute stage of Chagas’ disease has very mild and nonspecific symptoms that occur 4 to 8 weeks postinfection. As a result, very few infections are diagnosed at this stage. However, in ∼30% of individuals, infection manifests as a symptomatic chronic condition, although this can take many years to emerge (1). Most commonly, chronic disease is associated with cardiac dysfunction, and to a lesser extent, digestive tract pathologies. These sequelae result in the death of ∼12,500 people each year (2).

To date, benznidazole (2-nitroimidazole) and nifurtimox (5-nitrofuran) are the only approved drugs available for the treatment of Chagas’ disease. Prolonged treatment with these nitroimidazoles during the acute stage cures up to 70% of individuals; however, their efficacy decreases significantly in the chronic stage (3). Both therapies are associated with severe toxic side effects that can lead to the interruption or discontinuation of treatment in as many as 30% of cases (4, 5). It is clear that new, safe, effective, oral drugs that are suitable for short-course regimens are urgently required.

No new drugs have been developed for Chagas’ disease for more than 30 years. Recent clinical trials with posaconazole and the ravuconazole prodrug E1224 were disappointing, with relapse rates in between 70 and 90% of patients (6, 7), compared to to 6 to 30% failure for the benznidazole-treated arm of the study. The failure of both azoles, known to act via inhibition of lanosterol C_14_α-demethylase (CYP51), has led to a “root and branch” overhaul of the screening cascade and drug discovery approach for Chagas’ disease (8, 9). The principal goal of this process will be to vastly improve translation from *in vitro* and *in vivo* models for Chagas’ disease to the clinic.

Successful treatment of Chagas’ disease is now believed to require removal of every viable parasite within the infected patient. To complicate matters further, transiently dormant or persister forms of T. cruzi that are refractory to drugs acting via certain mechanisms of action (MoA) have recently been identified (10, 11). An additional barrier to the development of new drugs is the relative lack of robustly validated targets in T. cruzi. This has limited target-focused screening programs and led to a reliance upon phenotypic screening to identify start points for drug discovery. Phenotypic approaches have proven effective; however, a lack of information regarding the MoA or specific molecular target(s) of active compounds can hinder their downstream optimization in order to overcome pharmacokinetic and/or toxicity issues and to derive selectivity compared to human homologues. Furthermore, a comprehensive understanding of MoA can facilitate the deprioritization of compounds with unattractive or failed targets, such as CYP51, or those unable to clear all parasites and/or kill persister forms, as well as allowing the triaging of compounds targeting the same promising targets.

Here, we use a range of genetic and chemical proteomic approaches to determine the MoA of an arylsulfonamide compound demonstrating promising *in vitro* activity against T. cruzi. Our comprehensive studies reveal that this compound principally targets the T. cruzi proteasome, binding at the interface between the β4 and β5 subunits that catalyze chymotrypsin-like peptidase activity. Using thermal proteome profiling, we also confirm that this compound interacts with a secondary target, malic enzyme, albeit this interaction does not appear to drive potency. The implications of developing compounds with this MoA as anti-chagasic drugs in the future are discussed.

## RESULTS

### Arylsulfonamide compound demonstrating promising activity against *T. cruzi.*

**FIG 1:**
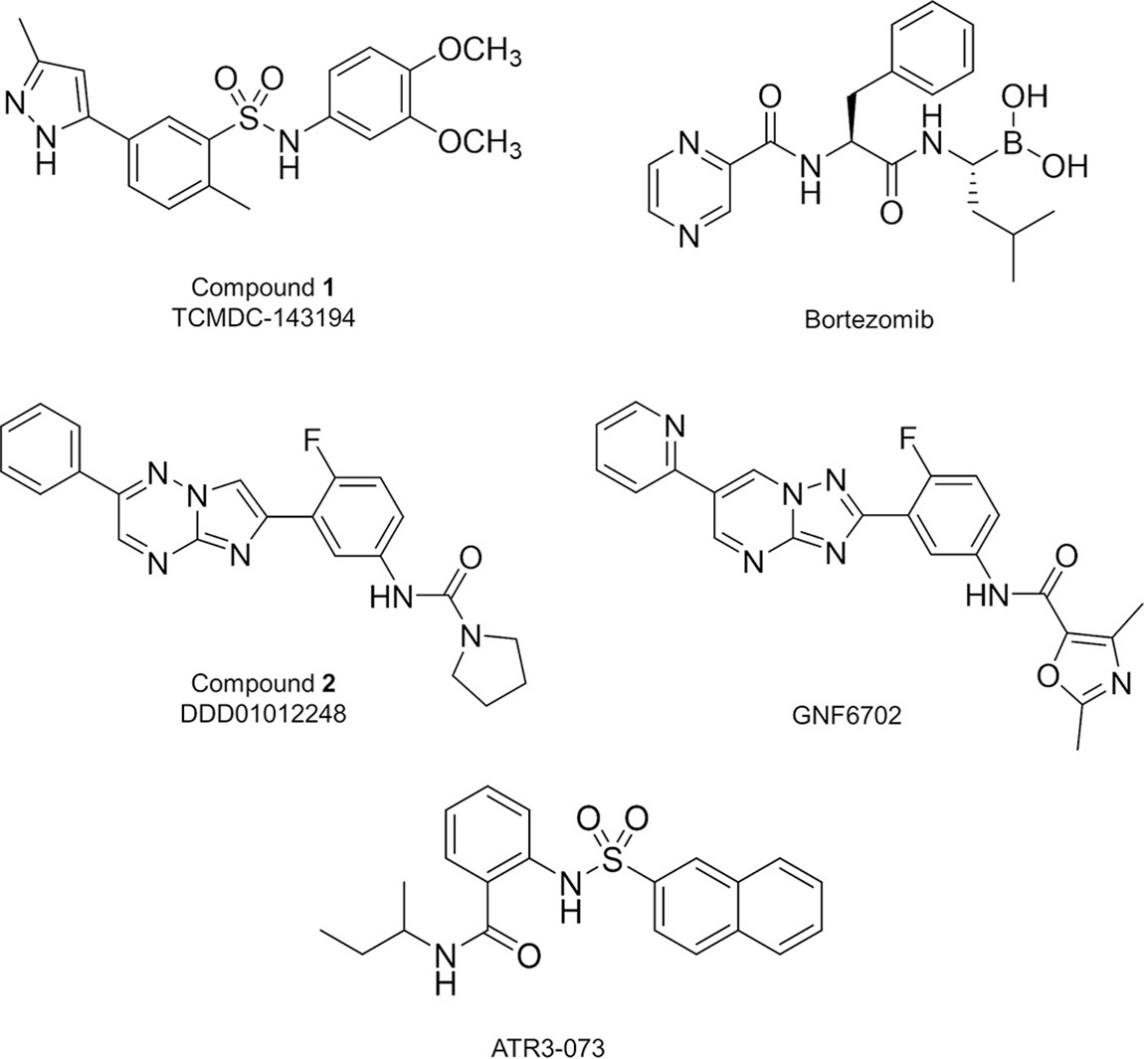
Structures of compounds used in this study. These compounds include TCMDC-143194, an *N*-aryl arylsulfonamide that originates from GSK’s kinetobox library (12); the anticancer drug bortezomib, a peptidic boronic acid; the compound DDD01012248, a close analogue of the *Leishmania* clinical candidate and proteasome inhibitor GSK3494245 (16); the imidazotriazine GNF6702, a pan-active inhibitor of the kinetoplastid proteasome developed by Novartis (20); and the triazolopyrimidine ATR-073, a proposed inhibitor of T. cruzi malic enzyme (cytosolic) (27).

High-throughput screening of GSK’s 1.8M compound library against L. donovani, T. cruzi, and T. brucei resulted in the identification of a significant number of compounds active against these parasites (12). Among these hits, TCMDC-143194 was found to be moderately active against all three kinetoplastids (compound 1, Fig. 1), with EC_50_ values ranging between 0.1 and 8 μM against the mammalian stages of these parasites (Table 1). Bearing in mind the paucity of well-validated molecular targets for Chagas drug discovery and the fact that compound 1 does not act through inhibition of CYP51 (CYP51 pIC_50_, 4.4 [12]), we proceeded with target identification studies predominantly in T. cruzi.

**TABLE 1:** Compound 1 potency^*a*^

Organism	Type	Mean compound 1 EC_50_ (μM) ± SD
T. cruzi	Epimastigote	3 ± 0.08
	Intra-Vero	1.3 ± 0.2
L. donovani	Promastigote	0.1 ± 0.005
	Axenic amastigote	0.7 ± 0.1
	Intramacrophage	8.0^*b*^
T. brucei	BSF	0.3 ± 0.013
Mammalian	HepG2	23.0 ± 3.0

a All EC_50_ values represent the weighted means for at least three biological replicates (*n *≥* *3), with each biological replicate comprised of two technical replicates.

b Intramacrophage data are from Peña et al. (12).

### Resistance generation followed by whole-genome sequencing.

**FIG 2:**
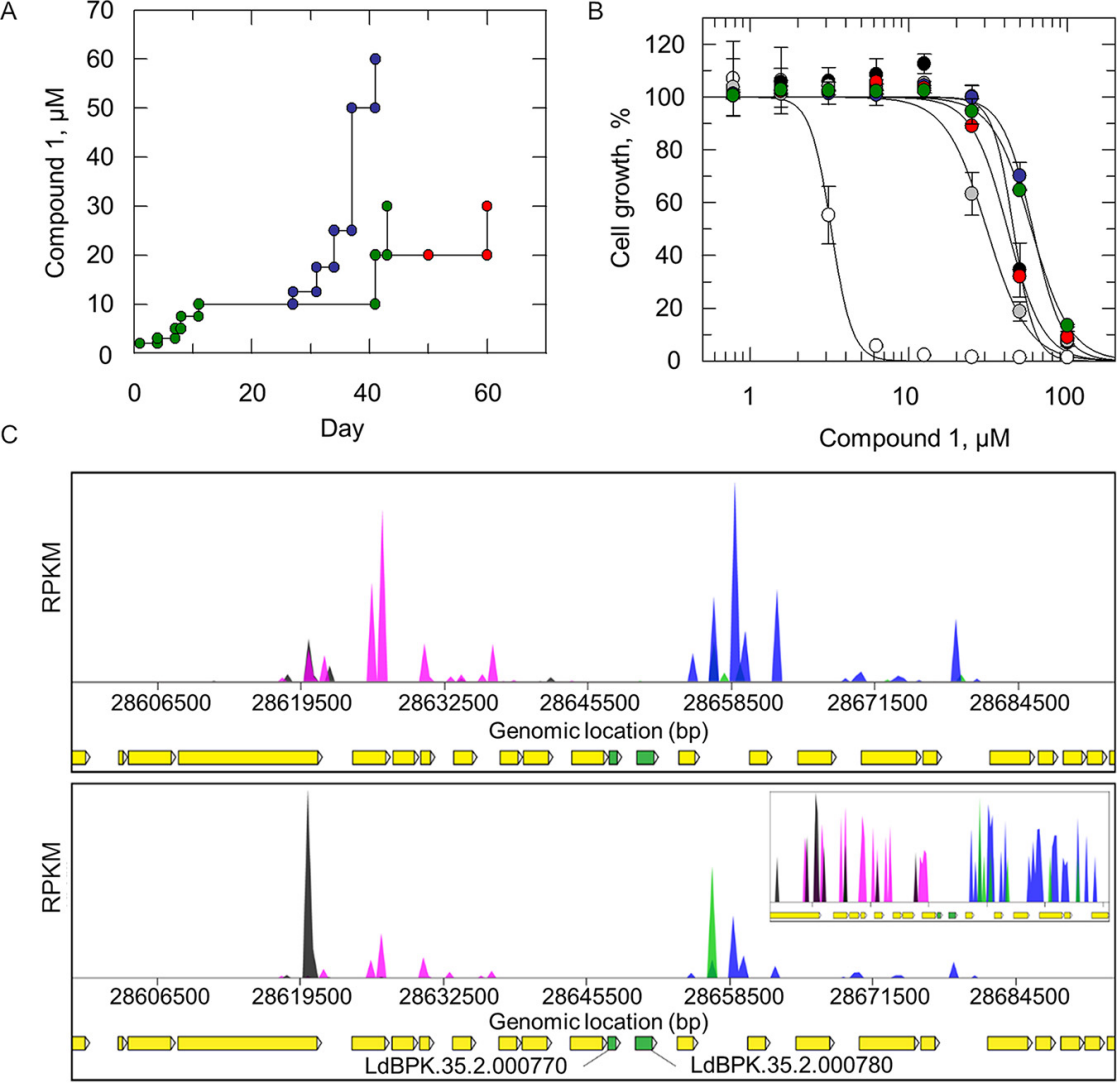
Target deconvolution studies with compound 1. (A) Schematic representation of the generation of compound 1-resistant cell lines in T. cruzi epimastigotes. Each passage of cells in culture (circles) is indicated with cell lines 1 to 5 indicated in black, gray, blue, red, and green, respectively. (B) EC_50_ values for compound 1 were determined for WT (white circles) and cloned resistant cell lines I-V (black, gray, blue, red, and green circles, respectively). These curves are the nonlinear fits of data using a two-parameter EC_50_ equation provided by GraFit. An EC_50_ value of 0.7 ± 0.01 μM was determined for compound 1 against WT promastigotes. EC_50_ values for resistant clones I to V were 23 ± 4, 16 ± 0.3, 13 ± 1, 11 ± 1, and 14 ± 6 μM, respectively. These EC_50_ curves and values are from one biological replicate, comprised of two technical replicates. Collated data sets reporting the weighted means ± the SD of multiple biological replicates are summarized in Table S4. (C) Genomewide map indicating cosmid library hits from screening of compound 2 (upper panel) and compound 1 (lower panel). Primary “hits” on chromosome 35 are indicated in green (LdBPK.35.2.000770 and LdBPK.35.2.0007800), with surrounding genes indicated in yellow. The blue/pink and black/green peaks indicate independent cosmid inserts in different orientations. (Lower panel inset) Focus on barcodes flanking LdBPK.35.2.000770 and LdBPK.35.2.0007800.

Our first step toward determining the MoA of compound 1 was to select T. cruzi epimastigote cell lines resistant to this arylsulfonamide. Starting at 3 μM (∼1 × EC_50_), five independent clonal lines of compound-susceptible parasites were exposed to stepwise increasing levels of compound 1 for 40 to 60 days, until they were routinely growing at concentrations equivalent to 10× to 20× the established EC_50_ value (Fig. 2A). The five independently generated resistant cell lines were cloned by limiting dilution, and clones were assessed for susceptibility to compound 1. The resulting clones were between 9- and 21-fold less sensitive to compound 1 than was the wild-type (WT) parental clone (Fig. 2B and Table 2). In each case, the resistance demonstrated by each clone was stable over 20 passages in culture in the absence of compound.

**TABLE 2:** Collated EC_50_ data for WT, resistant, and transgenic T. cruzi epimastigote cell lines^*a*^

Cell line	Mean EC_50_ (μM) ± SD^*b*^			
	Compound 1	Compound 2	GNF6702^*c*^	Bortezomib
Wild type	3.3 ± 0.08	0.04 ± 0.001	0.3 ± 0.02	0.2 ± 0.004
RES I	61 ± 1 (18)	4 ± 0.08 (100)	>10	0.2 ± 0.004 (1)
RES II	54 ± 1 (16)	5 ± 0.2 (125)	>10	0.3 ± 0.01 (1)
RES III	61 ± 0.7 (18)	5 ± 0.4 (125)	>10	0.2 ± 0.08 (1)
RES IV	31 ± 2 (9)	0.1 ± 0.01 (2.5)	>10	0.6 ± 0.05 (2.5)
RES V	70 ± 2 (21)	0.8 ± 0.03 (20)	10 ± 0.2 (33)	0.2 ± 0.08 (1)
β5^D225N-OE^	27 ± 1 (8)	2 ± 0.1 (58)	>10 (>28)	0.2 ± 0.008 (1)
Res I-β5^OE^	13 ± 0.8 (4)	0.3 ± 0.01 (6.5)	2 ± 0.3 (6)	0.2 ± 0.005 (1)
β4^F24L/I29M^	>50 (>15)	>10 (>250)	>10 (>28)	0.2 ± 0.005 (1)
ME^OE^	5 ± 0.11 (1)			

a All EC_50_ values represent the weighted means for at least three biological replicates (*n *≥* *3), with each biological replicate comprised of two technical replicates.

b The fold change versus the WT is indicated in parentheses where applicable.

c Solubility issues >10 μM.

Genomic DNA recovered from the five resistant clones was analyzed by whole-genome sequencing (WGS). Sequence reads were aligned to both T. cruzi Dm28c or Sylvio X10 reference genomes and compared to the wild-type parental clone (the data are summarized in Tables S2 and S3 in the supplemental material). Analysis of single nucleotide polymorphisms (SNPs) of resistant lines RES I to RES III identified a homozygous mutation (D^225^N) in the β5 subunit of the proteasome (C4B63_48g131). In addition, preexisting heterozygosity at position 54 (I/T) within β5 in the parental cell line became homozygous for Thr in all resistant lines. In cell line RES 5, a homozygous SNP (I^27^T) was also identified on the β4 subunit of the proteasome. Of the five compound 1-resistant clones, only RES IV, the least resistant of the five lines (9-fold), bore no mutations in any subunits of the proteasome. Instead, RES IV maintains a homozygous SNP (V^253^L) in an ABCG-like transporter (C4B63_63g86), a homologue of a *Leishmania* transporter with an established link to drug resistance (13, 14), as well as a heterozygous SNP (H^605^Q), on mitochondrial DNA polymerase I protein D. Relatively few copy number variations (CNV) were observed in resistant clones in comparison to the parental cell line. Extra copies of the proteasome regulatory ATPase subunit 5 (C4B63_76g37) and subunit 1 (C4B63_76g43) were identified in RES II, III, and IV, with both genes encoded on the same contig.

### Screening of compound 1 against a genomewide overexpression library.

As a parallel approach to identify the molecular target, compound 1 was screened against our genomewide overexpression library in the closely related kinetoplastid parasite Leishmania donovani (15). The principle underpinning this gain-of-function screen is that elevated levels of a drug target can result in resistance to the corresponding drug by increasing the pool of functional protein or by reducing free drug through binding. L. donovani promastigotes were transfected with a pooled population of cosmids containing genomic DNA fragments of between 35 and 45 kb. The final transfected library provides a >15-fold genome coverage with 99% of *Leishmania* genes represented. The library was selected with 300 nM compound 1 (equivalent to the EC_99_ in promastigotes) for 7 days and for a further 14 days at 600 nM. After compound selection, cosmids maintained by the “resistant” parasite population were harvested and analyzed by next-generation sequencing. Mapping of overexpressed inserts to the L. donovani LV9 and BPK281 assembled genomes revealed that 80% of all mapped reads aligned to a single region on chromosome 35 (Fig. 2C; see also Tables S4 and S5). This 63.3-kb region encodes 14 designated open reading frames in total. However, only two genes were flanked by all opposing barcodes: a CBS domain-containing protein (LdBPK.35.2.000780), also annotated as the γ subunit of an AMP-activated protein kinase, and the proteasome-activating protein PA28 (PA28; LdBPK.35.2.000770). Genomewide overexpression library screening with the established proteasome inhibitor compound 2 (compound 7 [16]), a close analogue of the clinical candidate for visceral leishmaniasis GSK3494245 (16, 17), confirmed that parasites “resistant” to this proteasome inhibitor were also found to overexpress PA28 (Fig. 2C; see also Table S5). Indeed, compound 2-resistant L. donovani promastigotes, generated through *in vitro* selections and subsequently found to bear a G^197^S mutation within the gene encoding the β5 proteasome subunit, demonstrate considerable (260-fold) cross-resistance to compound 1 (see Table S6). These data, alongside our WGS analysis, strongly suggest that, like compound 2, compound 1 is a proteasome inhibitor in both L. donovani and T. cruzi.

### Target validation.

The proteasome is a key component of the ubiquitin-proteasome protein degradation system and plays a crucial role in numerous cellular processes, including protein turnover and cell signaling (18). In eukaryotes, the proteasome consists of a central 20S cylindrical core flanked by two regulatory complexes (19S). The canonical 20S unit is comprised of two outer (α) and two inner (β) polypeptide rings. Three of the β-type subunits are responsible for chymotrypsin-, trypsin-, and caspase-like catalytic activities. The proteasome is a well-exploited target in drug discovery for a variety of indications, including cancer, inflammation, and a number of infectious diseases (19). A number of recent studies have illustrated the utility of the proteasome as a viable drug target in kinetoplastids (16, 20–22). Indeed, GSK3494245 and LXE408 (16, 22), inhibitors of the chymotrypsin-like activity of the *Leishmania* proteasome are being clinically assessed for use in the treatment of visceral leishmaniasis. These studies confirm the feasibility of selectively inhibiting of the kinetoplastid proteasome and the value of this molecular target for drug discovery.

We next sought to interrogate the role of the proteasome in the MoA of compound 1. Like compound 1, compound 2 is active against both T. cruzi and L. donovani. Virtually all of our compound 1-resistant clones, with the exception of RES IV (2.5-fold resistant), demonstrated considerable cross-resistance to this established proteasome inhibitor (20- to 127-fold; Table 2). In addition, the broad-spectrum anti-kinetoplastid proteasome inhibitor GNF6702 (20) demonstrated similar levels of cross-resistance against RES I to V clones, while there was no evidence of cross-resistance to the classical proteasome inhibitor bortezomib, used in the treatment of multiple myeloma, mantle cell lymphoma, and a number of other cancers (23) (Table 2). GNF6702, GSK3494245, and analogues are known to target the same allosteric binding site at the interface of the β4/β5 subunits of the proteasome resulting in the inhibition of chymotrypsin-like activity. Collectively, our data suggest that compound 1 targets this same allosteric binding site rather than the bortezomib binding pocket in the active site of β5.

The impact of the β5 D^225^N mutation, identified in the majority of our resistant clones, was examined further. T. cruzi epimastigotes overexpressing the mutated version of the β5 subunit were generated, with elevated levels of this mutated protein confirmed by quantitative RT-PCR (see Fig. S1). Epimastigotes overexpressing β5 D^225^N were found to be 8-fold less sensitive to compound 1 than wild-type parasites. These transgenic parasites also demonstrated considerable resistance to both compound 2 and GNF6702 (Table 2). Overexpression of β5^WT^ in the RES I clone, which bears the D^225^N mutation, partially reverted the resistance phenotype of this cell line to all three compounds. CRISPR-Cas9 was used to engineer specific mutations in the β4 subunit (F^24^L and I^29^M) previously shown to confer resistance to GNF6702 (20). CRISPR-edited epimastigotes were refractory to compound 1, compound 2, and GNF6702 at all of the concentrations tested, once again linking the mechanism(s) of action/resistance of compound 1 with that of established proteasome inhibitors. Furthermore, these data strengthen our hypothesis that compound 1 exploits the same allosteric binding site at the interface of the β4/β5 subunits of the proteasome.

The *in vitro*-selected, CRISPR-edited, and overexpressing T. cruzi cell lines were also assessed against compound 1, GNF6702, and fexinidazole (control compound) as amastigotes within Vero cells (see Table S7). The response to compound treatment and the resistance/cross-resistance profiles of these intracellular amastigotes closely mimicked that seen with their respective epimastigote cell lines. These data are consistent with compound 1 inhibiting the function of the proteasome in the clinically relevant, mammalian stage of T. cruzi.

### Inhibition of proteasome activity.

Initially, the impact of compound 1 on the chymotrypsin-like proteolytic activity of the proteasome was assessed using a commercially available indirect enzyme-based luminescent assay. In this assay, activity is monitored in proteasome-enriched T. cruzi epimastigote lysates using Suc-LLVY-aminoluciferin as a substrate (16). Unfortunately, the data produced by this assay were unreliable with regard to compound 1, with IC_50_ values ranging from 0.9 to >15 μM reported. Based on these data and the statistics associated with the assay, it became clear that compound 1 directly interferes with the assay. Thus, an alternative route to determine the impact of this arylsulfonamide compound on T. cruzi proteasome function was required.

**FIG 3:**
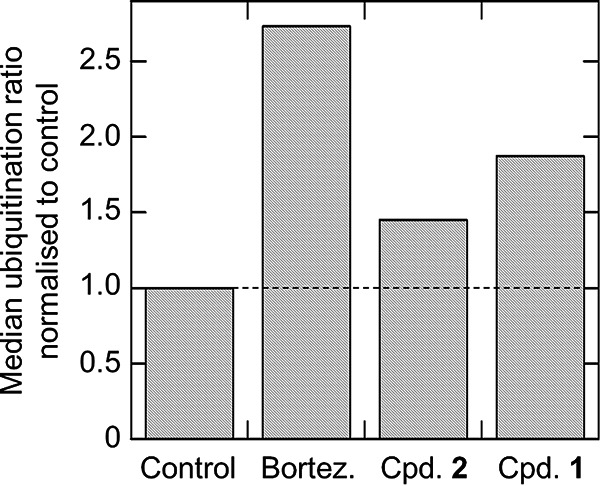
Relative levels of ubiquitinylated proteins in compound-treated and untreated T. cruzi epimastigotes. LC-MS/MS was used to quantify ubiquitinylated proteins recovered from T. cruzi epimastigotes pretreated (8 h) with bortezomib, GNF6702, or compound 1 at concentrations equivalent to 3× their respective EC_50_ values. The abundances of ubiquitinylated proteins relative to the levels in control cultures exposed to DMSO for 8 h are shown. The data are from one biological replicate.

In cells where the function of the proteasome has been compromised, there is a concomitant build-up of ubiquitinated proteins earmarked for degradation. Here, ubiquitinated peptides were recovered from the lysates of epimastigotes pretreated with bortezomib, compound 1, or compound 2 over 8 h at concentrations equivalent to 3× their respective EC_50_ values. Control cultures were treated for the same period of time in the presence of dimethyl sulfoxide (DMSO). Enrichment of ubiquitinated proteins was achieved by immunoprecipitation using magnetic beads conjugated to an antibody specific for the remnant of ubiquitinated lysines following digestion with trypsin and/or LysC. We used liquid chromatography-tandem mass spectrometry (LC-MS/MS) to calculate the ubiquitination ratio of each sample by dividing the reporter intensity of ubiquitinated proteins by the reporter intensity of total protein (see Table S8). As expected, the accumulation of ubiquitinylated proteins was highest in epimastigotes treated with bortezomib (2.7-fold higher than in the DMSO-treated control), which is known to inhibit all three catalytic activities of the proteasome (Fig. 3). The buildup of ubiquitinated proteins in cells treated with GNF6702, an established inhibitor of the chymotrypsin-like activity of the proteasome, was clearly evident but more modest (1.45-fold higher than for the control). Similarly, ubiquitinated proteins accumulated to levels 1.9-fold higher in compound 1-treated parasites compared to those recovered from DMSO-treated control cells. These data are entirely consistent with our hypothesis that compound 1 acts principally as an inhibitor of the T. cruzi proteasome.

### Identification of a secondary target.

Thermal proteome profiling (TPP) can be used as an effective and unbiased approach to demonstrate compound-target engagement. It is based on the principle that binding of a drug to its protein target can significantly alter the thermal stability of that protein (24). Here, T. cruzi epimastigote lysates were treated with compound 1 (10× the established EC_50_ value) or DMSO vehicle. Aliquots of each lysate were then incubated at designated temperatures (33 to 69°C) and then, for each temperature, insoluble (denatured) proteins were removed. The resulting soluble protein samples were reduced, alkylated, and digested with trypsin prior to derivatization with tandem mass tags. Pooled peptides were fractionated by high-pressure liquid chromatography and analyzed by LC/MS-MS prior to identification and quantitation. The melting points of identified proteins were then established using the TPP software package. Full melt curves were established for 6,771 proteins, representing 39.4% coverage of the T. cruzi proteome. The data for the top 20 proteins demonstrating a thermal shift in the presence of compound 1 in two separate replicate experiments are summarized in Tables S9 and S10.

**FIG 4:**
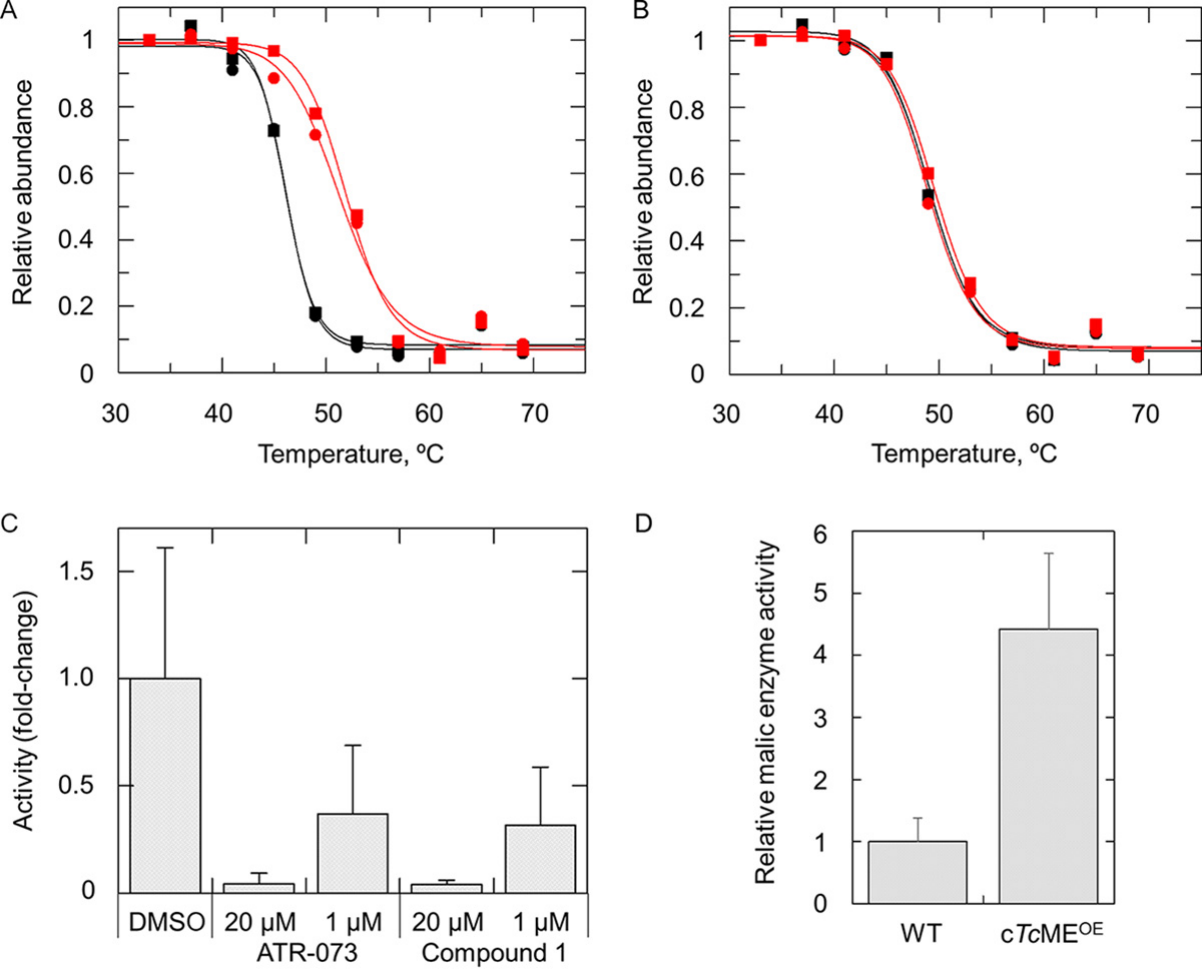
Interrogation of T. cruzi ME as a potential target of compound 1. (A and B) TPP melt curves for c*Tc*ME (A) and m*Tc*ME (B) after incubation with compound 1 (red) or vehicle (0.1% DMSO, black). Data from technical replicates (circles and squares) are shown, and the mean shift in melting temperature (Δ*T_m_*) for c*Tc*ME in this biological replicate was 5.5°C. (C) Effects of ATR-073 and compound 1 preincubation on the ME-dependent production of NADPH in clarified lysates of T. cruzi epimastigotes after the addition of malate. The data represent the enzyme activities relative to DMSO-treated control lysates and are expressed as the means ± the SD from >3 biological replicates. (D) Relative ME activities in wild-type and c*Tc*ME^OE^ cell lysates. The data represent the enzyme activities relative to the wild type and are expressed as the means ± the SD from >3 biological replicates.

While TPP has proven effective in a number of our MoA studies to date (15, 25), experience indicates that it is less effective in cases where the molecular target is part of a large multisubunit complex such as the proteasome. Indeed, analysis of our two independently generated TPP data sets failed to identify any subunits of the proteasome as targets of compound 1 (see Tables S9 and S10). The only target candidate with significantly increased thermal stability in the presence of compound 1 across both data sets was the cytosolic T. cruzi malic enzyme (c*Tc*ME; C4B63_28g106) (26). Individual melting curves revealed that the thermal stability of c*Tc*ME increased by 8.8°C (mean Δ*T_m_*) in experiment 1 and by 5.5°C in experiment 2 (Fig. 4A; see also Tables S9 and S10). In contrast, compound 1 had no impact on the thermal stability of the mitochondrial version of this enzyme (m*Tc*ME; Fig. 4B).

Malic enzyme (ME) catalyzes the oxidative decarboxylation of malate to pyruvate with the concomitant reduction of NAD(P)^+^ to NAD(P)H. In light of our TPP studies, we next sought to determine whether the binding of compound 1 to c*Tc*ME inhibits enzymatic activity. The ME-dependent production of NADPH was monitored in clarified lysates of T. cruzi epimastigotes at 340 nm after the addition of malate. In the first instance, this assay was validated using ATR-073, an established inhibitor of c*Tc*ME (27). In keeping with previous studies (27), preincubation of lysates with 20 μM ATR-073 resulted in a 96% reduction in ME activity, while pretreatment with 1 μM reduced ME activity by 63%. Similarly, preincubation of lysates with 20 μM and 1 μM concentrations of compound 1 inhibited ME activity by 96 and 68%, respectively (Fig. 4C). These data confirm compound 1 as an inhibitor of c*Tc*ME. It should be noted that ATR-073 and compound 1 are structurally similar, both being substituted *N*-phenyl aryl sulfonamides, this common moiety constituting the pharmacophore of this chemical series.

In order to probe the role of c*Tc*ME inhibition in the MoA of compound 1, a clonal cell line overexpressing this enzyme was generated. Elevated levels of c*Tc*ME in transgenic parasites relative to the wild type were confirmed by label-free MS quantitation (see Fig. S2). In addition, using the previously described spectrophotometric assay, we found the ME activity in c*Tc*ME^OE^ lysates to be 4.4-fold higher than in comparable wild-type lysates (Fig. 4D), confirming that overexpressed ME is enzymatically active. However, overexpression of c*Tc*ME had little to no effect on the potency of compound 1, and this was also the case for c*Tc*ME-overexpressing amastigotes in Vero cell assays (see Table S7), as well as c*Ld*ME-overexpressing L. donovani promastigotes (see Table S6). Epimastigotes overexpressing c*Tc*ME also remained just as susceptible to ATR-073 as wild-type epimastigotes, perhaps suggesting that ME is not the primary target of this compound (see Table S11). It should also be noted that careful analysis of WGS data from our T. cruzi compound 1-resistant clones identified no CNV or SNP associated with c*Tc*ME, nor did screening of our genomewide overexpression identify c*Tc*ME as a hit. We next investigated the possibility that ATR-073 may actually inhibit the T. cruzi proteasome. Using an established luciferase-based biochemical assay (21), this compound had no effect on the chymotrypsin-like activity of the proteasome at concentrations up to and including 3.3 μM (see Fig. S3A). At concentrations above this threshold ATR-073 began to interfere directly with the assay. In addition, cell lines resistant to compound 1 and bearing mutations in the β4 and β5 subunits of the proteasome demonstrated no cross-reactivity to ATR-073 (see Fig. S3B). Collectively, these data suggest that, despite the structural similarities between ATR-073 and compound 1, they likely interact with different molecular targets with T. cruzi.

### Docking studies.

**FIG 5:**
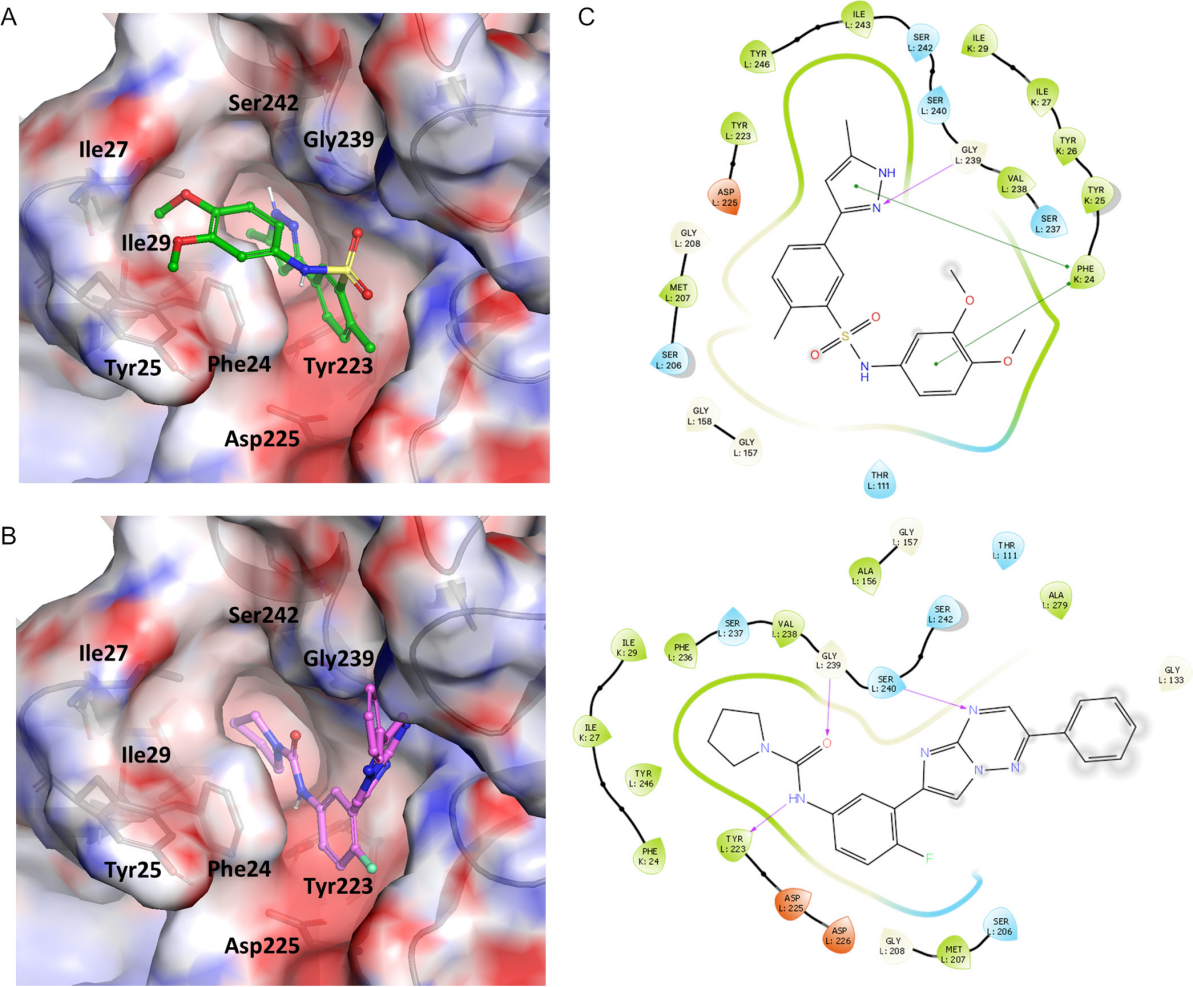
Compound 1 binding hypothesis. (A) Best-scoring binding pose for compound 1 (green) in the T. cruzi homology model of the GSK3494245 binding site at the interface of the β4/β5 subunits of the proteasome. (B) Best-scoring binding pose for compound 2, consistent with the pose observed in the cryo-EM structure of L. tarentolae proteasome with the close analogue GSK3494245 (PDB 6QM7). (C) Two-dimensional ligand interaction diagram based on the best-scoring docking pose for compounds 1 and 2. Amino acids shaded in green are considered hydrophobic, those in blue are charged positive, those in red are charged negative, and glycine is shaded yellow. Gray diffuse rings indicate solvent-exposed residues.

With the aim of defining the binding site of compound 1 and understanding the role of mutations in compound 1 resistance, a homology model of the T. cruzi β4 and β5 proteasome subunits was generated. This model was based on the L. tarentolae orthologue structure (PDB 6QM7) complexed with GSK34944245 (16). The L. tarentolae β4/β5 proteasome subunits share 78% overall sequence identity with their counterparts in T. cruzi (see Fig. S4). Indeed, the sequence identity of the GSK3494245 binding site is even greater, with only one of the 26 amino acids within 5 Å of the ligand differing (S^132^ of the β5 subunit in T. cruzi is T^122^ in L. tarentolae). Since there are two tautomeric forms of the compound 1 pyrazole, both were docked into the model. The best docking poses of both tautomers occupy the same region as GSK3494245 in the L. tarentolae cryoEM structure (16). The pyrazole moiety of compound 1 occupies the hydrophobic pocket formed by F^24^, I^27^, and I^29^ residues of the β4 subunit and Y^223^, V^238^, and Y^246^ from β5. It also establishes a hydrogen bond with the backbone nitrogen of G^239^ in β5 (or S^242^ side chain, depending on the tautomer). In this binding pose, compound 1 stacks with F^24^ from the β4 subunit, and the central phenyl ring is in close proximity to the β5 side chains of D^225^ and D^226^, while the sulfonamide points toward solvent, directing the di-metoxy phenyl moiety into a groove defined by β4 residues Y^25^, Y^26^, and I^27^ side chains (Fig. 5). Previous studies have shown that β5 residues D^225^ and D^226^ play an important role on the recognition of GSK3494245 by establishing long-range electrostatic interactions with a positively charged patch resulting from the unevenly distributed electrons of the ligand (17). Analysis of the electrostatic potential (ESP) surface of compound 1 suggests a similar scenario where a moderately electron-deficient area on the side of the pyrazole and central phenyl rings, possibly accentuated by the electron withdrawing effect of the sulfonyl amide, establishes a favorable electrostatic interaction with the side chains of the β5 residues Y^223^, D^225^, and D^226^ (see Fig. S5). This is consistent with the reduction in affinity observed for compound 1 and GSK3494245 in the presence of the mutation D^225^N in the β5 subunit, where the removal of the negatively charged side chain partially disrupts this favorable interaction. Based on the suggested mode of binding, I^27^ from β4 plays a critical role in defining the subpockets where the pyrazole and di-metoxy phenyl moieties of compound 1 bind, and its mutation would likely disrupt the binding of the ligand, as evidenced by the resistance-conferring I^27^T mutation.

## DISCUSSION

Multiple orthogonal genetic, biochemical, and proteomics approaches identify the proteasome as the primary molecular target of an arylsulfonamide compound with potential for anti-chagasic drug discovery. The proteasome has long been considered a viable, theoretical drug target in trypanosomatids (28), largely based on the enhanced susceptibility of these parasites to established proteasome inhibitors relative to mammalian cells (21, 29). Several recent studies have validated this assumption, with two proteasome inhibitors now in clinical trials for use in the treatment of visceral leishmaniasis (GSK3494245 and LXE408). Both compounds are structurally related and exploit the same binding pocket at the interface of the β4/β5 subunits of the L. donovani proteasome (16, 20, 22). Binding to this site inhibits the chymotrypsin—but not the trypsin—and caspase-like activities of the *Leishmania* proteasome. Inhibitors that target this pocket demonstrate impressive selective inhibition of the parasite versus the human proteasome, and this has been attributed to sequence variability in the narrow hydrophobic pocket (16), while the corresponding pocket in the human proteasome is also more open, shallow, and solvent exposed. Despite sharing no structural similarity with either LXE408, GSK3494245, or analogues, we present here multiple lines of evidence that compound 1 targets the equivalent binding pocket in the T. cruzi proteasome (Fig. 5B and C). Thus, compound 1 represents a new scaffold capable of binding at the proteasome β4/β5 subunit interface that may represent a suitable starting point for future antichagasic drug discovery.

The structure of compound 1 closely resembles a series of compounds found to inhibit c*Tc*ME in a target-based high-throughput screen (27). While our data indicate that compound 1 directly binds to c*Tc*ME and inhibits enzymatic activity, this inhibition does not appear to drive potency in epimastigotes or intracellular amastigotes. However, we cannot rule out the possibility that c*Tc*ME inhibition in parasites where the function of the proteasome is also compromised may contribute to compound 1 potency. To our knowledge, there is no direct evidence that either ME isoforms are essential in T. cruzi. In the related kinetoplastid T. brucei, RNAi studies in the insect procyclic stage of the parasite indicate that, while mME is essential in standard cell culture conditions, the cytosolic isoform is dispensable (30). Establishing whether cME is essential in T. cruzi will be important in understanding its precise role, if any, in the MoA of compound 1 and will also determine whether this enzyme has any value for future Chagas’ disease drug discovery.

Subpopulations of nondividing intracellular amastigotes have been observed in both *in vitro* and *in vivo* T. cruzi infections. These “persister” parasites retain the ability to differentiate into infectious trypomastigote forms that can reactivate infection. Importantly, nonreplicating amastigotes are refractory to treatment with existing trypanocidal compounds, including the frontline therapy benznidazole (10). These observations have led many to associate dormancy with treatment failure and to suggest that only compounds capable of killing dormant forms should be pursued (11). Based on their reduced ability to express reporter proteins (10), it is assumed that persister amastigotes exist in a significantly reduced metabolic state concurrent with reduced protein synthesis. It is tempting to hypothesize that alongside reduced protein synthesis, the requirement to degrade proteins via the proteasome will be similarly reduced in persisters, leading to a decreased susceptibility to proteasome inhibitors. However, the proteasome inhibitor GNF6702 dosed twice daily at 10 mg kg^−1^ matched the efficacy of benznidazole in a mouse model of infection (20). All but one of the eight treated mice had no detectable parasites in blood, colon, or heart tissue, even after 4 weeks of immunosuppression. It should also be noted that a modified treatment regimen where higher doses of benznidazole were given in pulses over prolonged periods resulted in sterile parasitological cure of multiple mouse models of infection (31). This high-dose, extended-time protocol is believed to improve benznidazole efficacy by challenging the stochastic, time-limited nature of dormant parasites in T. cruzi infections. These exemplary studies provide a template for the effective use of promising compounds that potentially have a modest impact on persister parasites. Assessing the efficacy of known proteasome inhibitors, including compound 1, via this modified protocol should be a priority.

In conclusion, we have identified here a novel pharmacophore capable of inhibiting the T. cruzi proteasome. Future studies should focus on optimizing the drug-like properties of this promising compound in order to assess its efficacy in mouse models of Chagas’ disease.

## MATERIALS AND METHODS

### Compounds.

Compound 1 was kindly provided by GlaxoSmithKline. The malic enzyme inhibitor ATR-073 was purchased from MolPort. DDD01012248 (16) and GNF6702 (20) were kindly provided by the Drug Discovery Unit, University of Dundee. Bortezomib was purchased from Sigma-Aldrich. The structures of all compounds used in this study are shown in Fig. 1.

### Cell lines and culture conditions.

The clonal Leishmania donovani cell line LdBOB (derived from MHOM/SD/62/1S-CL2D) was grown as either promastigotes or axenic amastigotes in media specific for each developmental stage, as previously described (32). T. cruzi epimastigotes from the Silvio strain (MHOM/BR/78/Silvio; clone X10/7A [33]) were grown at 28°C in RTH/FBS (RPMI 1640 medium supplemented with Trypticase, hemin, HEPES, and 10% heat-inactivated fetal calf serum (FCS) [Fisher Scientific]) (34). Bloodstream form T. brucei “single marker” S427 (T7RPOL TETR NEO) were cultured in the presence of G418 (15 μg ml^−1^) at 37°C in HMI9-T media in the presence of 5% CO_2_. HepG2 cells (ECACC 85011430) were obtained from European Collection of Authenticated Cell Culture (ECACC). Cells were maintained in full growth medium (minimal essential medium [MEM] with GlutaMAX (Thermo Fisher), supplemented with 1% MEM nonessential amino acids (Sigma) and 10% heat-inactivated FBS and cultured at 37°C in the presence of 5% CO_2_. Cells were passaged twice weekly by detaching adherent cells with 0.05% trypsin/EDTA (Sigma) and diluted into fresh media. Cells were never grown beyond 80% confluence. Vero cells (African green monkey kidney cells; ECCAC 84113001) were maintained at 37°C, 5% CO_2_ in Dulbecco modified Eagle medium (DMEM; Lonza) supplemented with 10% FCS and subcultured every 2 days. T. cruzi metacyclic trypomastigotes were obtained from late-log-phase epimastigote cultures (initial inoculum, 10^6^ ml^−1^) after ca. 7 to 10 days at 28°C in RTH/FBS. Trypomastigote-rich cultures were incubated with Vero cells overnight at 37°C and 5% CO_2_ in DMEM/10% FCS. The following day, extracellular parasites were removed by washing the Vero cell monolayer three times. Infected monolayers were maintained at 37°C and in 5% CO_2_, DMEM/10% FCS was replaced every 48 h until the trypomastigotes reemerged from the Vero cells (9).

### Drug sensitivity assays.

To examine the effects of test compounds on the growth of T. cruzi epimastigotes, mid-log-phase parasites were seeded into 96-well plates at a cell density of 5 × 10^5^ cells ml^−1^. Cells were exposed to test compounds over a range of concentrations (2-fold serial dilutions). Cells were incubated for 4 days, and then 20 μl of 2.5 mM resazurin was added to each well, before measuring the fluorescence (excitation, 528 nm; emission, 590 nm), following a further 24 h of incubation. Data were processed using GRAFIT (Erithacus Software) and fitted to a two-parameter equation, where the data are corrected for background fluorescence, to obtain the effective concentration inhibiting growth by 50% (EC_50_):

$$y=\frac{100}{1+{\left(\frac{\left[I\right]}{{\text{EC}}_{50}}\right)}^{m}}.$$

In this equation, [*I*] represents the inhibitor concentration, and *m* is the slope factor. Experiments were repeated at least two times, and the data are presented as means plus the standard deviations.

L. donovani promastigote (35), axenic amastigote (25), and T. brucei bloodstream form (36) drug sensitivity assays were carried out as previously described. HepG2 monolayers were washed twice in PBS, detached with 0.05% trypsin/EDTA, and then diluted with fully supplemented MEM growth medium. Cells were pelleted at 80 × *g* for 5 min and resuspended in fully supplemented growth medium. Cells were seeded into 96-well plates (5 × 10^4^/well) and allowed to adhere prior to exposure to test compounds. Plates were incubated at 37°C in the presence of 5% CO_2_ for 72 h. After incubation, resazurin (20 μl of a 2.5-mg/ml^−1^ stock solution) was added to each well, followed by incubation for a further 2 h prior to fluorescence being read as described above.

Drug sensitivity assays against T. cruzi-infected Vero cells were carried out as previously described (37); however, in this instance, assays were carried out in 96-well plates. Data were processed using GRAFIT (Erithacus software) and fitted to a two-parameter equation, as described above.

### Cosmid library screening.

The construction of our cosmid-based genomewide overexpression library in L. donovani and strategy used to screen the library have been described in detail previously (25). For compound 2 (Fig. 1), the library was selected for 2 days at 8 nM, 2 days at 16 nM, and a further 12 days at 30 nM prior to harvesting and analysis. For compound 1 (Fig. 1), the library was selected for 7 days at 300 nM and for a further 14 days at 600 nM. The associated data sets have been deposited with the European Nucleotide Archive under accession number PRJEB39157.

### Resistance generation.

Compound-resistant cell lines were generated by subculturing a clone of wild-type T. cruzi epimastigotes in the continuous presence of compound 1. Starting at sublethal concentrations, drug concentrations in five independent cultures were increased in a stepwise manner. When parasites were able to survive and grow in concentrations of compound 1 equivalent to >20× the established EC_50_ value, the resulting cell lines were cloned by limiting dilution in the presence of compound. Five clones (RES 1 to RES 5) were selected for further biological study.

### Whole-genome sequencing analysis.

Genomic DNA was isolated from WT and resistant clones using a standard alkaline lysis protocol. DNA was sequencing on an Illumina 4000 machine by the Beijing Genomics Institute. Sequences reads were aligned to the T. cruzi Sylvio X10 (v39) or T. cruzi Dm28c 2018 genome sequence (v46; tritrypdb.org) alongside the maxi-circle sequence (FJ203996.1; NCBI). Reads were aligned using Bowtie2 using the settings “–very-sensitive” and SAMtools software. SNPs and indels were called using SAMtools (mpileup) and BCFtools (38), where the overall quality score was >100 compared to the wild-type starter clone. Chromosome and gene copy number variation (CNV) analysis, as well as visualizations, was performed using Artemis. Median read counts of the wild-type and resistant clones were used to normalize copy number. The associated data sets have been deposited with the European Nucleotide Archive under accession number PRJEB39157.

### Lysate production for thermal proteome profiling.

T. cruzi (X10/7 strain) mid-log-phase epimastigotes (∼1 × 10^10^) were harvested by centrifugation (1,912 × *g*, 15 min, 4°C) and washed with ice-cold PBS (1,912 × *g*, 5 min, 4°C); finally, the cell pellet was resuspended in 8 ml of ice-cold lysis buffer (1 mM EDTA, 1 mM dithiothreitol [DTT], 100 μM TLCK [*N*α-*p*-tosyl-l-lysine chloromethyl ketone], and 1× Roche EDTA-free COmplete protease inhibitor cocktail in 50 mM potassium phosphate buffer [pH 7.4]). The cell suspension was submitted to three freeze-thaw cycles in a dry ice/ethanol bath to biologically inactivate the parasites and then submitted to cell disruption (Constant Systems, UK) at 30 kpsi. The resulting lysate was centrifuged (100,000 × *g*, 20 min, 4°C), the supernatant was collected, and the protein concentration was determined using a Bio-Rad protein assay.

### TPP assays.

The lysate concentration was adjusted to 2.5 mg ml^−1^ with lysis buffer and then 2 × 2-ml aliquots were incubated at room temperature for 30 min in the presence of test compound at 20 μM (equivalent to 10× EC_50_) or vehicle (0.1% DMSO). Each 2-ml aliquot (drug and vehicle treated) was divided into 10 × 100-μl aliquots in 0.5-ml thin-walled PCR tubes, followed by incubation at a designated temperature (33, 37, 41, 45, 49, 53, 57, 61, 65, or 69°C) for 3 min, followed in turn by incubation at room temperature for 3 min before each sample was placed on ice. Each aliquot was centrifuged (100,000 × *g*, 20 min, 4°C), the supernatants were harvested, and the protein concentration was assessed.

### TPP sample processing, analyses, and data processing.

All aspects of sample processing, peptide and protein identification and quantitation, and target identification were carried out as previously described (15). However, in this instance, proteins were identified by searching the MS and MS/MS data for the peptides against T. cruzi proteome Dm28c 2018 version 50 (https://tritrypdb.org/tritrypdb).

### Generation of overexpression constructs.

Malic enzyme (*TcME*, C4B63_28g106), β5 (*Tcβ5^WT^*, C4B63_48g131), and *β5^D225N^* (*Tcβ5^D225N^*) overexpression constructs were assembled by inserting synthetic versions of each gene (GeneArt; Invitrogen) into the pTREX vector via EcoRI and XhoI sites (39). The L. donovani malic enzyme (LdBPK_240780.1) overexpression construct was assembled by inserting a synthetic version of the gene (GeneArt) into the pIR1SAT vector via BglII sites. All overexpression constructs were sequenced in-house to confirm their accuracy. T. cruzi overexpression constructs were linearized with NheI prior to transfection.

### Generation of CRISPR-Cas9 edited *T. cruzi* cell line.

T. cruzi proteasome β4 subunit base editing was achieved by mixing a Cas9 expression plasmid (10 μg), a specific sgRNA template, a repair template (40 μg), and T7 RNA polymerase and then transfecting the mixture into T. cruzi epimastigotes. Briefly, pRPa^T7Cas9^ was assembled by replacing the ribosomal DNA (rDNA) promoter in pRPa^Cas9^ (40) with a T7 promoter. pRPa^Cas9^ was digested with NheI and HindIII to remove the rDNA promoter and replaced by the following sequence containing the T7 promoter GCTAGC**TAATACGACTCACTATAGGG**CCCTGCACGCGCCTTCGAGTTTTTTTTCCTTTTCCCCATTTTTTTCAACTTGAAGACTTCAATTACACCAAAAAGTAAAATTCACAAGCTT (the restriction sites are underlined, and the T7 promoter sequence is in boldface). The remaining sequence corresponds to an untranslated region upstream of the *procyclin* gene that was removed with the rDNA promoter and needs to be reinstated for the correct processing of Cas9 mRNA. The sgRNA template was generated by annealing and end-filling the following FTcProtB4g and R-uni-scaf oligonucleotides: FTcProtB4g (TAATACGACTCACTATAGGG**CATCAAGATCATGGACACGG***GTTTTAGAGCTAGAAATAGCAAG*; the T7 promoter is underlined, the specific gRNA target sequence is in boldface, and the partial gRNA scaffold sequence is in italics) and R-uni-scaf (GCACCGACTCGGTGCCACTTTTTCAAGTTGATAACGGACTAGCCTTATTTTAACTTGCTATTTCTAGCTCTAAAAC), the full sgRNA scaffold sequence. These oligonucleotides (both at 2 μM) were annealed at 50°C and end filled at 72°C for 15 s (5 cycles) in the presence of HiFi polymerase (Roche). The repair template was FTcProtB4g (TAGCAGCAGCAGGGCTGAATGCCTT**A**TACTATATTAAAAT**G**ATGGATACAGAAGATAAGGTCACGCAGTTGGATTCCC; nonsynonymous edits are in boldface, and synonymous changes are underlined [one of these disrupts the Cas9 protospacer-adjacent motif to prevent further DNA breaks]). The three DNA components were combined, ethanol precipitated, resuspended in 10 μl of dH_2_O, and—following the addition of 5 μl of T7 RNA polymerase—electroporated into T. cruzi epimastigotes, as described below. Cells were allowed to recover for 24 h and then selected with GNF6702 at 1.5 μM. Resistant cells were subcloned, DNA was extracted from independent subclones, and a specific portion of the T. cruzi proteasome β4 gene (TCSYLVIO_007432) encompassing the edited region was amplified using the PCR primers FB4-PCR (ATGTCGGAGACAACCATTGCTTTTC) and RB3-PCR (CCATGTAGTACAAGTGTGGTCC). The PCR products were Sanger sequenced in-house.

### Transfection of *L. donovani* and *T. cruzi* transgenic cell lines.

Mid-log-phase epimastigotes (2 × 10^7^ cells in total) were transfected with 5- to 10-μg portions of overexpression constructs using a Human T-Cell Nucleofector kit and an Amaxa Nucleofector electroporator (program U-033). After transfection, the cells were allowed to recover for 16 to 24 h before the appropriate drug selection (200 μg ml^−1^ G418). L. donovani transgenic cell lines were generated as previously described (41) and selected with nourseothricin (100 μg ml^−1^). In all cases, cloned cell lines were generated by limiting dilution, maintained in selective medium, and removed from drug selection for one passage prior to experiments.

### RT-qPCR.

RNA was harvested from mid-log-phase epimastigotes (1 × 10^8^ cells total) using an RNeasy minikit (Qiagen) according to the manufacturer’s instructions. The remaining DNA was degraded from samples using an RNase-Free DNase set (Qiagen). Quantitative RT-PCR was performed with 100 ng of total RNA using a Luna Universal One-Step RT-qPCR kit (New England Biolabs) with the following reaction conditions: 10 min at 55°C for the reverse transcription step, followed by a denaturation step of 1 min at 95°C and then by 40 cycles of 10 s at 95°C, with a final extension for 30 s at 60°C. Relative quantification was established using the reference glyceraldehyde-3-phosphate dehydrogenase (*GAPDH*) gene. Primers (listed below) were designed using the Primer3Plus website. The levels of each transcript in the overexpression cell lines were normalized to the wild type using the ΔΔ*C_T_* method. Two independently transfected clones for each construct were used, and the statistical significance was measured using a Student unpaired *t* test.

### Label-free quantification.

The relative protein abundance in the WT versus the overexpressing cell lines was established as previously described (25). In this instance, proteins were identified by searching the protein sequence database containing L. donovani BPK282A1 or T. cruzi Dm28c annotated proteins (downloaded from TriTrypDB 46 [http://www.tritrypdb.org]).

### Proteasome assays. (i) Luminescence.

The effect of inhibitors on the chymotrypsin-like activity of the T. cruzi proteasome was assessed by using a luminescence-based assay, as previously described (21).

### (ii) Treatment with proteasome inhibitors and lysate preparation.

T. cruzi epimastigotes in the logarithmic growth phase (3 × 10^6^ cells ml^−1^) were incubated for 8h with bortezomib (1.8 μM), GNF6702 (2.9 μM) (Fig. 1), or compound **1** (24 μM), equivalent to 8× the EC_50_ values of each compound. Controls were incubated in the presence of diluent (DMSO). Cells were harvested by centrifugation (1,912 × *g*, 15 min, 4°C) and washed with ice-cold PBS (1,912 × *g*, 5 min, 4°C); finally, the cell pellets were resuspended in 1.5 ml of ice-cold lysis buffer (1 mM EDTA, 1 mM DTT, 100 μM TLCK, and 1× Roche EDTA-free cOmplete protease inhibitor cocktail in 50 mM potassium phosphate buffer [pH 7.4]). Cell suspensions were submitted to three freeze-thaw cycles in a dry ice/ethanol bath to biologically inactivate the parasites and then lysed using a OneShot cell disruptor (Constant Systems) at 30 kpsi.

### (iii) Sample processing and enrichment.

Cell lysates were centrifuged (100,000 × *g*, 20 min, 4°C), supernatants were collected, and the protein concentrations were determined by using a standard Bio-Rad protein assay. Aliquots (1.1 mg) were reduced by incubating with 25 mM Tris(2-carboxyethyl)phosphine hydrochloride (TCEP) for 10 min at 37°C and alkylated by incubating with 25 mM iodoacetamide for 1 h at room temperature in the dark. Samples were then precipitated by incubation with 10% (vol/vol) trichloroacetic acid for 3 h at −20°C, followed by three washes with ice-cold acetone. Protein pellets were resuspended in 100 mM triethylammonium bicarbonate and digested with 40 μg Lys-C for 6 h, followed by 40 μg of trypsin overnight (25:1 protein/enzyme ratio). Protein digests were dried via evaporation, and the digestion efficiency was checked by mass spectrometry. Small aliquots of each sample (9% of the total sample) were kept for total proteome analysis, with the remainder submitted to enrichment. Ubiquitinated proteins were enriched using a PTMScan HS Ubiquitin/SUMO Remnant Motif (K-ε-GG) kit (Cell Signaling Technologies) according to the manufacturer’s recommendations. This kit contains antibodies conjugated to magnetic beads that specifically recognize the remnant of ubiquitinated lysines after digestion with trypsin and/or LysC. This remnant consists of a Gly-Gly (diGly) motif bound to the ε-amine of lysine through an isopeptide bond. Lysine ubiquitination results in a miscleavage, since tryptic enzymes are not able to cut after ubiquitinated lysines. Briefly, dried digests were resuspended in HS IAP bind buffer and incubated with magnetic beads conjugated to antibodies recognizing the anti-K-ε-GG motif for 2 h at 2°C. The beads were then washed with HS IAP wash buffer and water to remove unbound peptides. Bound peptides were eluted by incubation in agitation with IAP elution buffer (0.15% trifluoroacetic acid) for 10 min. Eluates were dried under vacuum and labeled with the TMTs 126, 127N, 127C, and 128N using a TMT 10plex isobaric tagging kit (Thermo Scientific) and then used to label control, GNF6702, bortezomib, and compound 1 samples, respectively, in parallel with their respective (total) proteome samples. After 1 h, the reaction was quenched by the addition of 5% hydroxylamine for 15 min, and then the four samples were pooled and vacuum-dried. The two pooled samples (the total proteome and the enriched fraction) were desalted using the Pierce peptide desalting spin columns (Thermo), and the eluates were vacuum-dried.

### (iv) LC-MS/MS.

Analysis of peptides was performed on a Q-Exactive-HF (Thermo Scientific) mass spectrometer coupled to a Dionex Ultimate 3000 RS (Thermo Scientific). The following LC buffers were used: buffer A (0.1% [vol/vol] formic acid in Milli-Q water) and buffer B (80% [vol/vol] acetonitrile and 0.08% [vol/vol] formic acid in Milli-Q water). Aliquots of each sample (1 μl) were loaded at 5 μl/min^−1^ onto a trap column (100 μm × 2 cm, PepMap nanoViper C_18_ column, 5 μm, 100 Å; Thermo Scientific) equilibrated in 5% buffer B. The trap column was washed for 5 min at the same flow rate and then switched in-line with a Thermo Scientific, resolving C_18_ column (75 μm × 50 cm, PepMap RSLC C_18_ column, 2 μm, 100 Å). Peptides were eluted from the column at a constant flow rate of 300 nl/min^−1^ with a linear gradient from 5% buffer B (for fractions 1 to 10 [7% for fractions 11 to 20]) to 35% buffer B in 130 min and then to 98% buffer B at 132 min. The column was then washed with 98% buffer B for 20 min and reequilibrated in 5% buffer B for 17 min. Q-Exactive HF was used in data-dependent mode. A scan cycle comprised MS1 scan (*m/z* range from 335 to 1,800, with a maximum ion injection time of 50 ms, a resolution of 120,000, and an automatic gain control [AGC] of 3 × 106), followed by 15 sequence-dependent MS2 scans (with an isolation window set to 0.7 Da, a resolution at 60,000, a maximum ion injection time at 200 ms, and an AGC of 1 × 105). To ensure mass accuracy, the mass spectrometer was calibrated on the first day that the runs were performed.

### Protein search and data analysis.

MS data were analyzed using the software MaxQuant (https://maxquant.net/maxquant/; version 2.0.1.0). For the enriched fractions, reporter ion MS2 mode was selected using N terminus TMT10plex and carbamidomethyl (C) as fixed modifications, while oxidation (M), acetyl (protein N-term), lysine TMT10plex, DiGly, and a set of DiGly-lysine TMT10plex modifications were set as variable modifications. Proteins were identified by searching the MS and MS/MS data for peptides against Trypanosoma cruzi Dm28c proteome (TriTrypDB version 50, tritrypdb.org). Trypsin/P and LysC/P were selected as the digestive enzymes. For the total proteome samples, reporter ion MS2 mode was selected using the TMT-10plex labels on N terminus and lysine; carbamidomethyl (C) was set as fixed modification, while oxidation (M) and acetyl (protein N-term) were set as variable modifications. Protein abundance was calculated according to the normalized reporter ion intensities, which for the enriched fractions were calculated using only DiGly-modified peptides. The false discovery rate threshold for peptides and proteins was 0.01. Two missed tryptic cleavages were allowed in the global proteome samples, while three were allowed in the enriched fractions; the FTMS MS/MS mass tolerance was set to 10 ppm, and the ITMS MS/MS mass tolerance was set to 0.06 Da. The mass spectrometry proteomics data have been deposited to the ProteomeXchange Consortium via the PRIDE partner repository under the data set identifier PXD027524. Data were analyzed using the Perseus software (https://maxquant.net/perseus/, version 1.6.15.0) and RStudio (version 1.2.5033).

### Malic enzyme assays.

Logarithmic T. cruzi epimastigotes were harvested (1,690 × *g*, 10 min, 4°C), washed once in ice-cold phosphate-buffered saline, and resuspended at 2 × 10^9^ cells ml^−1^ in lysis buffer (10 mM phosphate buffer [pH 7.2], 10 mM EDTA, 5 mM benzamidine, 5 mM phenanthroline, 0.1 mM phenylmethylsulfonyl fluoride) containing 1 mg ml^−1^ digitonin, followed by incubation at 28°C for 10 min. The resulting lysate was then centrifuged (13,000 × *g*, 10 min, 4°C), and the supernatant was harvested and stored on ice. The protein concentration of this clarified lysate was determined by using a standard Bio-Rad protein assay.

The activity of malic enzyme was assayed as previously described (42), with minor modifications. T. cruzi clarified lysate (30 μl) was added to a reaction mixture (500 μl final volume) containing 50 mM Tris-HCl buffer (pH 7.6), 1 mM MnCl_2_, and 0.12 mM NADP^+^. Reactions were initiated by the addition of 5 mM l-malate. The reduction of NADP^+^ was monitored at 340 nm using a UV-2401 spectrophotometer (Shimadzu). To monitor the effect of test compounds on the activity of malic enzyme, compounds (1 and 20 μM) were preincubated for 10 min with the lysate in the reaction mixture. DMSO (0.2%) was used as control. All enzymatic activities were calculated as Δabs min^−1 ^mg^−1^ of protein.

### Homology model.

A model of the subunits β4 and β5 of the T. cruzi proteasome was generated using Modeller version 9.24 (https://www.salilab.org/modeller/) (43) based on the K and L chains of the L. tarentolae proteasome cocrystallized with compound GSK3494245 (PDB 6QM7). T. cruzi β4 (C4B63_13g138) and β5 (C4B63_48g131) sequences were aligned to the L. tarentolae template using the alignment 2D function in Modeller (see Fig. S2). A set of five models was generated with the GSK3494245 compound in the binding site, and the best ranked, based on Modeller scores, was chosen for docking calculations.

### Docking.

Ionization states and tautomers for compound 1 were assigned using LigPrep (Schrödinger) at the default pH range (7 ± 2). Pyrazole tautomerism indicated two equally populated tautomers and both were used for subsequent docking calculations. The T. cruzi model was refined and optimized using Protein Preparation Wizard: hydrogen bond networks were optimized for hydroxyls, thiols, and sidechain amide groups of the protein residues (Ser, Tyr, Cys, Asn, and Gln, respectively). Tautomers were evaluated for imidazole rings (His) and optimized. Protonation states of charged residues (His, Asp, Glu, Arg, and Lys) were evaluated. Optimization was carried out using OPLS3e forcefield and the VSGB implicit solvation model. A grid centered on the GSK3494245 ligand was generated for Glide XP docking (Schrödinger).

### Molecular electrostatic potential.

Electrostatic potential (ESP) maps were generated on the ligand binding pose using the DFT method in Jaguar at the B3LYP-D3/6-31+G(d,p) level of theory. ESP surfaces were represented using a 0.001 isovalue, and surfaces were mapped on the ESP using a rainbow color scale. Protein ESP was generated using the ESP surface panel in Maestro by solving Poisson-Boltzmann equations using the atomic partial charges of the protein residues (Schrödinger). The docking protocol XP was used at default settings, which include a postdocking minimization step.

### Data availability.

Genomics data sets from this study have been deposited with the European Nucleotide Archive under accession number PRJEB39157. Proteomics data from this study have been deposited to the ProteomeXchange Consortium via the PRIDE partner repository with the data set identifier PXD027524. All additional information data are available upon request from the corresponding author.
