# Supplementary material for: Identification of a Proteasome-Targeting Arylsulfonamide with Potential for the Treatment of Chagas’ Disease
Source: Antimicrob Agents Chemother. 2022 Jan 18;66(1):e01535-21. doi: 10.1128/AAC.01535-21 (PMC8765320; doi:10.1128/AAC.01535-21)

Supplementary information

Table S1: Summary of primers used in RT-qPCR.

**Primers**	**Sequences** (5' - 3')
TcGAPDH_F	GTGCGGCTGCTGTCAACAT
TcGAPDH_R	AAAGACATGCCCGTCAGCTT
TcMalic-Fw	ATAACATCTCCGCCAACGTC
TcMalic-Rv	AGTACACCGGCTTCCACATC
ProtB5qPCR-Fw	TGTGGGCTCAGGCTCTATCT
ProtB5qPCR-Rv	TTGCATGAAAAATGGAACGA

Table S2: Read number and fold coverage of whole genome sequencing analysis.

Table S3: Single nucleotide polymorphisms identified in open reading frames identified following of whole genome sequencing of compound **1**-resistant cell lines.

Table S4: RPKM and gene names of cosmid library 'hits' after selection with compound **1** (total region >5000 RPKM and >1 fragment).

Table S5: RPKM and gene names of cosmid library 'hits' after selection with compound **2** (total region >5000 RPKM and >1 fragment).

Table S6 – Collated EC_{50} data for WT, resistant and transgenic *L. donovani* cell lines.

Cell line	Compound 1 EC_{50} values, μM (fold change versus WT)
Wild-type	0.1 ± 0.005 (-)
Compound **2** RES III*	26 ± 4 (260)
c*Ld*ME^{OE}	0.1 ± 0.005 (=)

L. donovani* cell line resistant to compound **2 bearing a G197S mutation in the β5 subunit of the proteasome (12). All EC_{50} values represent the weighted mean ± standard deviation of at least three biological replicates (n ≥ 3) with each biological replicate comprised of two technical replicates.

Table S7 - Collated EC_{50} data for WT, resistant and transgenic *T. cruzi* cell lines in Vero cells.

Cell line	EC_{50} values, μM (fold change versus WT)		
	Compound 1	GNF6702	Fexinidazole
Wild-type	1 ± 0.2 (1)	0.2 ± 0.03 (1)	4 ± 0.8 (1)
RES 1	10 ± 5 (8)	16 ± 3 (85)	8 ± 1 (2)
RES 5	>50 (>42)	3 ± 0.4 (14)	4 ± 0.7 (1)
$β5^{OE}$	1 ± 0.2 (1)	0.2 ± 0.1 (1)	3 ± 0.3 (1)
$β5^{OE}$ rescue R1	3 ± 0.2 (2)	0.3* ± 0.1 (2)	4 ± 0.6 (1)
$β5^{D225N\text{-}OE}$	12 ± 1.4 (10)	> 5 (>25)*	3 ± 0.6 (1)
$β4^{F24L/I29M}$	>23 (>19)	> 1.5 (>8)	6 ± 0.6 (2)
ME^{OE}	0.8 ± 0.07 (1)	0.05* ± 0.01 (0.25)*	5 ± 1 (1)

All data represents the weighted mean ± standard deviation of three biological replicates with the exception of annotated values (*) which represent data from one biological replicate.

Protein ID	ΔT_m 1	p-value	ΔT_m 2	p-value	Protein name
C4B63_119g34	7.39	1.91E-06	3.89	0.001159	retrotransposon hot spot (RHS) protein
C4B63_11g96	-7.15	2.24E-07	-7.46	2.99E-05	protein kinase
C4B63_13g215	-5.52	0.009331	-3.59	0.138823	conserved hypothetical protein
C4B63_13g228	2.44	0.000813	3.17	0.138823	pre-mRNA-splicing factor ATP-dependent RNA helicase
C4B63_153g41	3.16	3.63E-06	2.00	0.075566	inositol 5-phosphatase 1(fragment)
C4B63_184g36	-4.95	0.041441	-4.16	0.013536	Vesicle-associated membrane protein 7
C4B63_188g44	-2.44	0.026678	-2.92	0.006095	conserved hypothetical protein
C4B63_218g24	2.67	0.000718	4.96	7.55E-07	Cullin family/Cullin protein neddylation domain containing protein
C4B63_22g269c	-2.86	0.05376	-3.81	9.12E-05	glutaredoxin
C4B63_26g233	-2.97	0.114299	-7.66	1.56E-05	mitochondrial DNA topoisomerase II
C4B63_28g106	8.60	4.28E-38	9.08	1.27E-15	malic enzyme
C4B63_297g18	2.10	0.041092	2.53	0.069381	conserved hypothetical protein
C4B63_2g455	4.59	5.05E-07	5.13	0.001469	Cytoplasmic dynein 2 heavy chain (DYNC2H1)
C4B63_2g691	-5.32	0.014042	-3.12	0.126048	30S Ribosomal protein S17
C4B63_328g5	-7.61	1.11E-08	-4.22	8.96E-06	conserved hypothetical protein
C4B63_41g242	-2.90	0.039256	-3.29	0.021793	conserved hypothetical protein
C4B63_42g60	-4.27	0.093074	-2.99	0.004584	amastin

C4B63_45g95	-2.64	0.005606	-5.82	5.46E-05	conserved hypothetical protein
C4B63_53g216	-2.98	0.095607	-4.15	0.00772	retrotransposon hot spot (RHS) protein
C4B63_61g142	2.25	0.024541	3.34	0.106747	conserved hypothetical protein

Table S8 - Top 20 hits identified by T_m analysis in biological replicate 1.

Protein ID	ΔT_m 1	*p*-value	ΔT_m 2	*p*-value	Protein name
C4B63_109g37	-9.66	8.7E-15	-12.47	6.09E-26	Gar1/Naf1 RNA binding region containing protein
C4B63_10g137	-2.43	0.005907	-3.25	0.141724	conserved hypothetical protein
C4B63_120g72	-7.25	1.15E-26	-10.99	1.81E-19	conserved hypothetical protein
C4B63_121g2	-7.14	5.15E-08	-9.76	2.04E-33	retrotransposon hot spot protein (RHS)
C4B63_12g292	-7.25	1.15E-26	-10.99	1.81E-19	conserved hypothetical protein
C4B63_14g113	-4.64	3.12E-10	-8.84	1.98E-09	conserved hypothetical protein
C4B63_158g46	5.23	0.003531	6.73	1.38E-09	retrotransposon hot spot protein (RHS)
C4B63_163g21	-8.95	1.13E-12	-9.86	3.86E-15	deoxyribose-phosphate aldolase
C4B63_16g205	-5.43	8.7E-15	-9.26	4.86E-13	retrotransposon hot spot (RHS) protein
C4B63_172g14	-6.42	1.81E-06	-11.54	9.52E-22	retrotransposon hot spot (RHS) protein
C4B63_17g1218c	7.00	0.001603	4.47	0.000788	conserved hypothetical protein
C4B63_20g223	-3.13	0.126456	-3.48	0.034656	SHQ1 protein
C4B63_20g307	4.53	3.23E-06	4.48	8.9E-09	conserved hypothetical protein
C4B63_226g19	5.23	0.003531	6.73	1.38E-09	retrotransposon hot spot protein (RHS)
C4B63_23g265	5.67	3.79E-10	2.89	6.55E-07	conserved hypothetical protein
C4B63_247g23	5.38	0.000609	4.09	4.46E-16	damage-specific DNA binding protein
C4B63_250g10	-6.31	3.02E-06	-7.27	2.86E-07	retrotransposon hot spot (RHS) protein

C4B63_28g106	5.60	2.08E-12	3.73	1.11E-06	malic enzyme
C4B63_2g415	3.27	4.37E-05	1.77	9.38E-06	conserved hypothetical protein
C4B63_31g213	2.57	0.003384	2.79	0.000783	conserved hypothetical protein

Table S9 - Top 20 hits identified by T_m analysis in biological replicate 2

Table S10 – Potency of an established ME inhibitor (ATR-073) against WT and ME^{OE} *T. cruzi* epimastigotes.

Cell line	ATR-073 EC_{50} values, μM (fold change versus WT)
Wild-type	34 ± 1
c*Tc*ME^{OE}	32 ± 2 (-)

All EC_{50} values represent the weighted mean ± standard deviation of at least three biological replicates (n ≥ 3) with each biological replicate comprised of two technical replicates.

Supplementary figures

Figure S1 - Quantitative RT-PCR confirming overexpression of mutated and wild-type versions of the β5 subunit of the proteasome in transgenic cell lines. The β5 subunit bearing a $D^{225}N$ mutation was overexpressed in wild-type parasites while the unmutated subunit was overexpressed in compound **1**-resistant cell line RES 1.

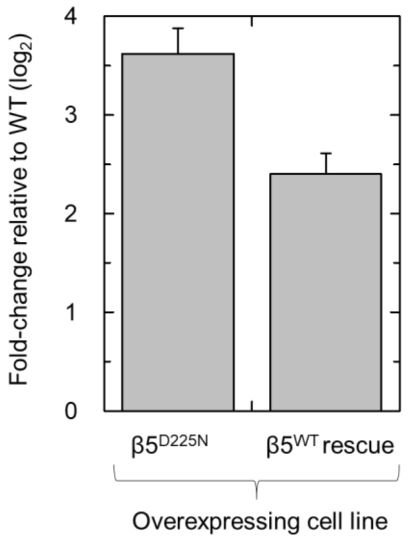

Figure S2 - Label-free proteomics quantitation. Relative protein levels in wild-type versus c*Tc*ME-overexpressing cell lines with c*Tc*ME indicated in red.

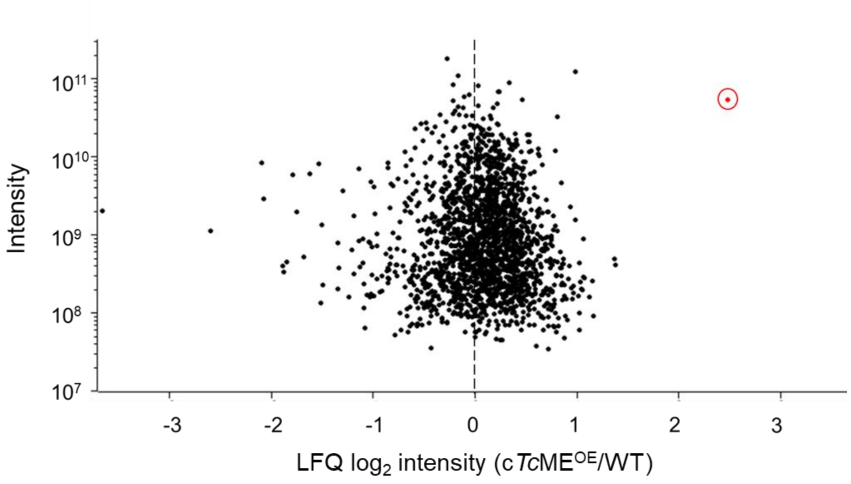

Figure S3 – Assessing the effect of ATR-073 on the *T. cruzi* proteasome. (A) Cell-free *T. cruzi* proteasome chymotrypsin-like activity concentration-response curves for ATR-073. At concentrations above 3.7 µM (indicated by a red line), ATR-073 began to interefere directly with the assay. Data are shown for 1 biological replicate (n = 3). The error bars represent SD. (B) EC_{50} values of 49 ± 29, 49 ± 1 and 51 ± 18 µM were established for ATR-073 against WT (open circles), $\beta 4^{F24L/I29M}$ (blue circles) and compound **2**-Res V cells, respectively. These EC_{50} values are from one biological replicate, comprised of three technical replicates.

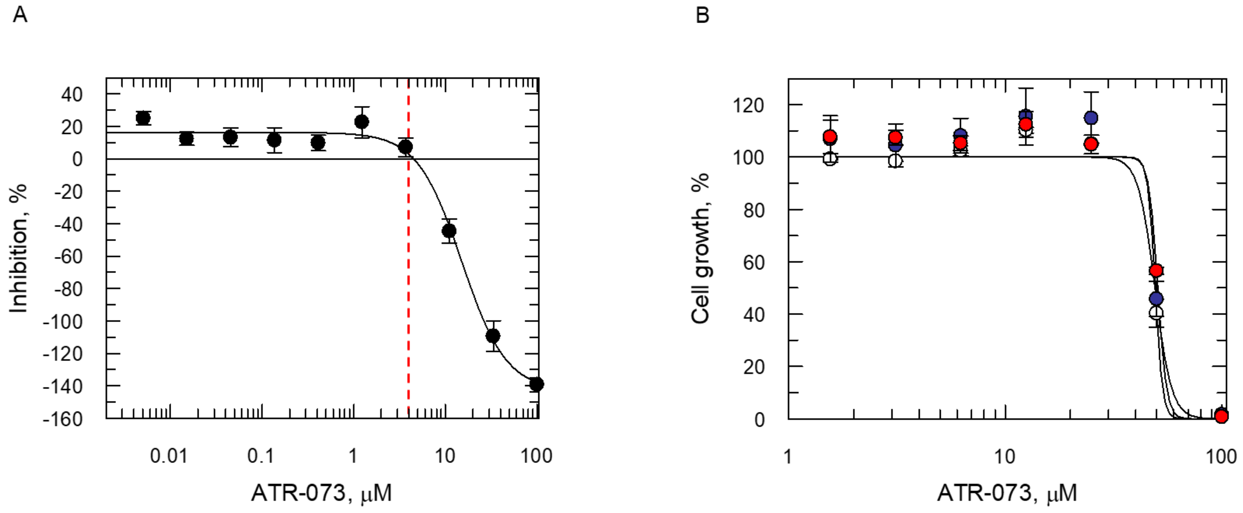

Figure S4 – Sequence alignment of β4 and β5 subunits of the proteasome. Amino acids within 5Å of the GSK3494245 binding site are identified in red boxes.

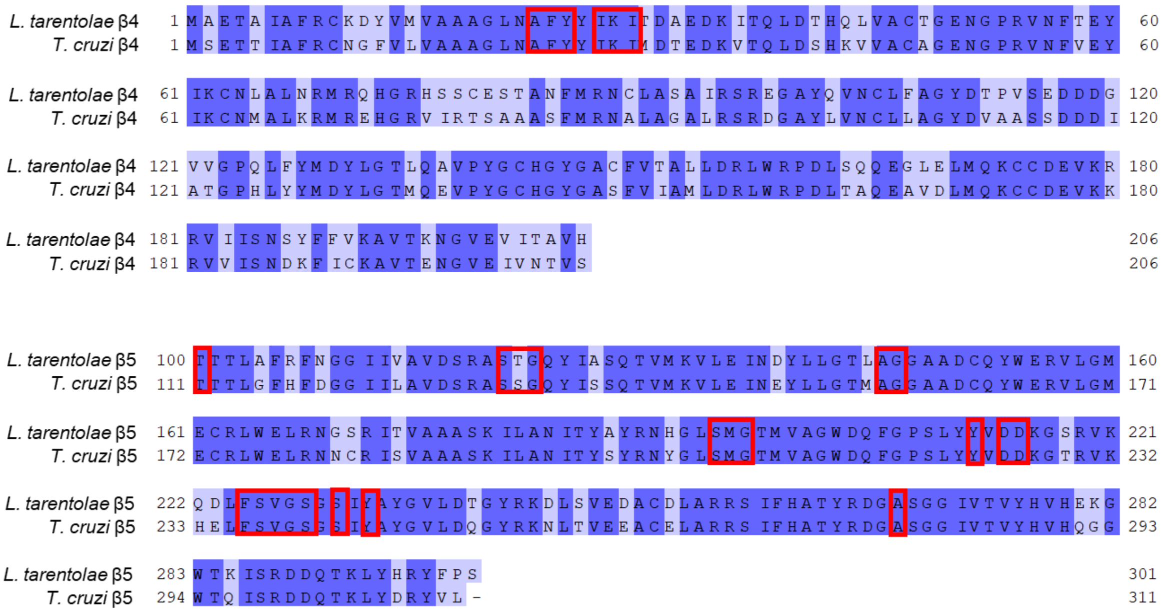

β4-β5 subunit	*L. tarentolae*	*T. cruzi*
L. tarentolae	100	98
T. cruzi	98	100

Figure S5 – Electrostatic potential representation of compound **1** (A), GSK3494245 (B), and their respective localisation at the binding site colored by protein electrostatic potential (C,D).

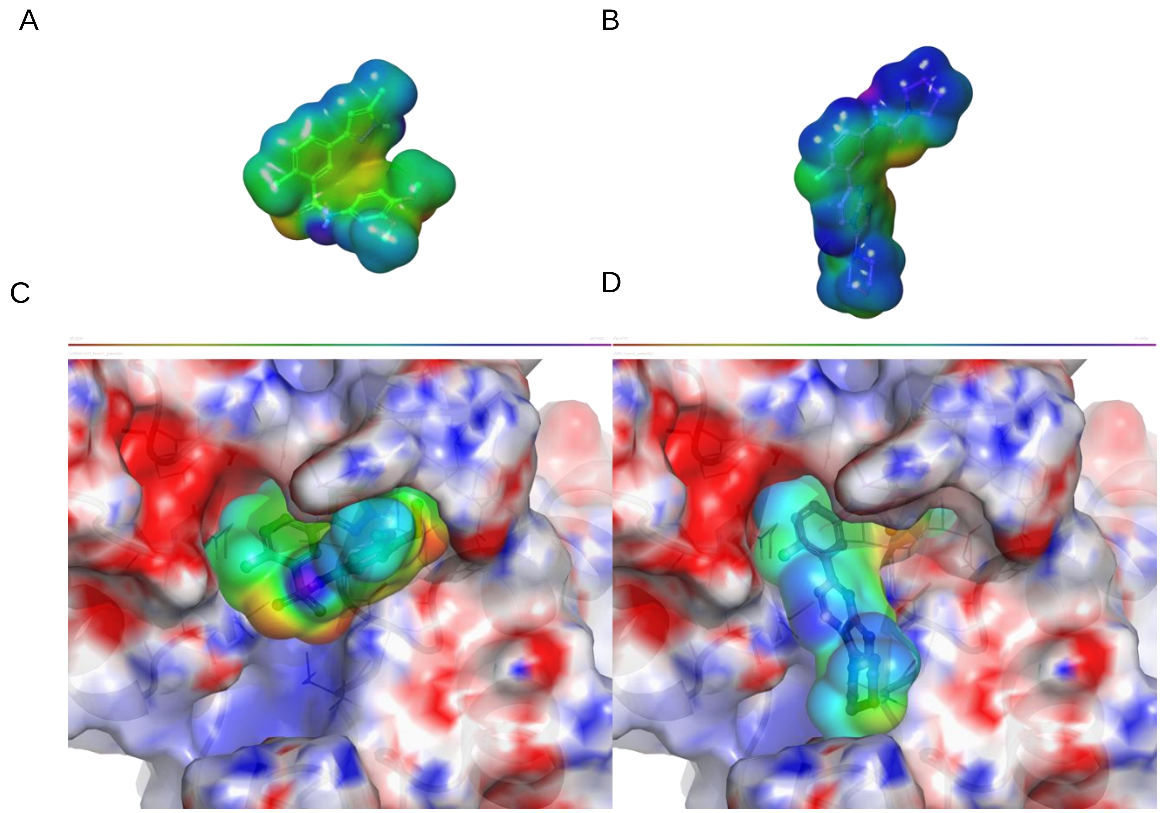

Supplement: Supplemental file 1 — Supplemental tables and figures. Download AAC.01535-21-s0002.pdf, PDF file, 0.6 MB [file aac.01535-21-s0002.pdf]
